# Circular RNAs function as ceRNAs to regulate and control human cancer progression

**DOI:** 10.1186/s12943-018-0827-8

**Published:** 2018-04-07

**Authors:** Yaxian Zhong, Yajun Du, Xue Yang, Yongzhen Mo, Chunmei Fan, Fang Xiong, Daixi Ren, Xin Ye, Chunwei Li, Yumin Wang, Fang Wei, Can Guo, Xu Wu, Xiaoling Li, Yong Li, Guiyuan Li, Zhaoyang Zeng, Wei Xiong

**Affiliations:** 1 The Key Laboratory of Carcinogenesis of the Chinese Ministry of Health, Xiangya Hospital, Central South University, Changsha, Hunan China; 2 The Key Laboratory of Carcinogenesis and Cancer Invasion of the Chinese Ministry of Education, Basic Medical Science School and Cancer Research Institute, Central South University, Changsha, Hunan China; 3 Hunan Key Laboratory of Nonresolving Inflammation and Cancer, Disease Genome Research Center, The Third Xiangya Hospital, Central South University, Changsha, Hunan China; 4 Department of Cancer Biology, Lerner Research Institute, Cleveland Clinic, Cleveland, OH USA

**Keywords:** Circular RNAs, ceRNA, Cancer, Gene expression regulation

## Abstract

Circular RNAs (circRNAs) are connected at the 3′ and 5′ ends by exon or intron cyclization, forming a complete ring structure. circRNA is more stable and conservative than linear RNA and abounds in various organisms. In recent years, increasing numbers of reports have found that circRNA plays a major role in the biological functions of a network of competing endogenous RNA (ceRNA). circRNAs can compete together with microRNAs (miRNAs) to influence the stability of target RNAs or their translation, thus, regulating gene expression at the transcriptional level. circRNAs are involved in biological processes such as tumor cell proliferation, apoptosis, invasion, and migration as ceRNAs. circRNAs, therefore, represent promising candidates for clinical diagnosis and treatment. Here, we review the progress in studying the role of circRNAs as ceRNAs in tumors and highlight the participation of circRNAs in signal transduction pathways to regulate cellular functions.

## Background

In 1976, researchers identified circular RNA (circRNA) in RNA viruses [1]. Then, in 1979, Hus [2] observed ring molecules, which are formed through the linkage of the 3′ and 5′ ends with a covalent bond, in HeLa cells under a microscope. However, these circRNAs without a characteristic tail were usually ignored during the separation process when using traditional RNA sequencing methods; thus, circRNAs were neglected until 1993, when their presence was confirmed in the human body [3]. Then, following improvements in experimental methods, the role of circRNA became increasingly clearer. circRNAs have features such as stability, sequence conservation, and expression specificity and exist in many cells [4]. Although many types of circRNAs have been discovered, their functions are still unclear. Two papers that elucidated the function of circRNA [2, 5] were published in 2013. Afterward, the field of circRNA research boomed. Recently, many studies have shown that circRNAs are critical in many diseases, especially in the progression of human cancer.

The ceRNA (competing endogenous RNA) hypothesis proposes a mechanism whereby various types of RNA control gene expression at the posttranscriptional level, which implies that mRNAs, the transcripts of pseudogenes, long non-coding RNAs (lncRNAs) [6–10], and circRNAs [11] affect the stability or the translation of target RNAs by competitively binding to the same microRNA (miRNA) [12–14].

circRNA, which plays a role in biological regulation primarily via gene regulation, is a type of non-coding RNA. circRNAs have extremely abundant binding sites for microRNAs (miRNA) and thus acts via absorption of miRNAs like a sponge [15]. circRNA can also act on other RNAs by base pairing [16]. In addition, circRNA can inhibit the activity of proteins by interacting with them [17]. Although circRNA represents a type of non-coding RNA, under certain conditions, it can act as a translation template for protein synthesis and produce functional proteins. Recently, studies have found that circRNAs can regulate the expression of host proteins, interact with RNA-binding proteins controlling transcription, play a role in transcriptional regulation in *cis*, and even regulate and control alternative splicing. However, most studies have shown that the ceRNA system represents the main means through which circRNA achieves its biological functions. This paper reviews the mechanism whereby circRNAs act as ceRNAs to control tumors.

### circRNA acts as ceRNA

#### ceRNA mechanism

The ceRNA hypothesis, a previously unknown pattern of regulating gene expression, was formally proposed by Salmena et al. [18] in 2011. The hypothesis states that miRNAs can affect the stability and translation of RNAs at the posttranscriptional level by binding to target genes, and conversely that RNAs can also affect miRNAs. Different varieties of ceRNAs have been discovered, including mRNAs, the transcripts of pseudogenes, lncRNAs, and circRNAs. All contain miRNA response elements (MREs) [11, 19–22]. circRNAs, as special endogenous non-coding RNAs, have become a new research focus among the ceRNA family after lncRNAs.

**Fig. 1:**
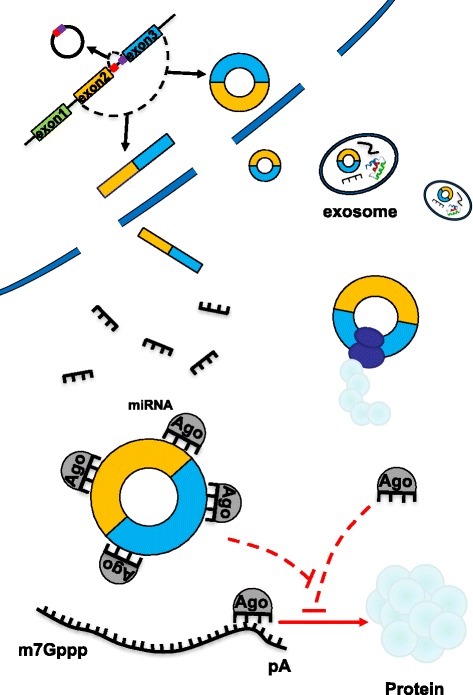
circRNAs serve as endogenous RNA competitors. A linear RNA precursor can be cis-spliced into linear RNA, and special sequences in the RNA precursor can combine to form a cycle to produce circRNAs. circRNAs act as endogenous RNA competitors to absorb microRNA (miRNA); as a result, the activity of the corresponding miRNA is reduced. miRNAs mainly act on mRNAs, and circRNA competition process blocks miRNAs from regulating the expression of target genes. This interaction between circRNAs and miRNAs has been shown to also occur in exosomes

RNA transcripts that have the same MREs can competitively inhibit the function of miRNAs, acting as an RNA sponge to block and inhibit miRNAs from binding to their target sites [18]; in addition, an RNA that has the same MREs can competitively bind miRNAs **(**Fig. 1). Thus, RNA molecules can reciprocally influence each other’s expression via miRNAs, which results in mutual regulation. Furthermore, the more similar miRNA types shared between two RNAs [23], the stronger their competitive relationship.

#### Factors affecting ceRNA activity

Recent studies have found that the activity of ceRNA is decided by a series of factors, including the abundance of miRNAs/ceRNAs, RNA editing, changes in the RNA 3′ UTR and RNA binding proteins (RBPs) [24].

Mukherji [25] proposed a hypothesis known as the “target threshold effect”: when the abundance of miRNAs and ceRNAs is nearly equal, the activity of ceRNAs is optimal. When the total level of transcription far exceeds miRNA levels, the role of ceRNA is substantially reduced because the amount of available miRNA is limited. Conversely, when the amount of miRNA is much higher than that of ceRNA, interactions are unlikely to occur because most transcripts are completely inhibited [25, 26]. Kumar et al. [27, 28] further demonstrated that the optimal ceRNA interactions occur when the levels of ceRNAs and miRNAs are similar.

RNA editing is a posttranscriptional gene regulation mechanism [29] that changes the genetic information in mRNA through the insertion, deletion, and substitution of nucleotides. Recent studies have shown that RNA editing affects the ceRNA system mainly in two ways. miRNA editing may affect the biosynthesis process, thus affecting the stability of the RNA molecules and even changing their roles and targets [30]. In addition, the editing of downstream RNA molecules may eliminate or produce a new miRNA binding site.

miRNAs mainly act on the 3′ UTRs of mRNAs and engage in mutual regulation. Therefore, a change in the 3′ UTR of an mRNA molecule will affect the ceRNA system. Mayer [31] found that compared with normal cells, cancer cells express higher amounts of mRNA subtypes with shorter 3′ UTRs. For transcripts with shorter 3′ UTRs, the number of miRNAs directly targeting these UTRs will decrease, and their ability to regulate other transcripts as ceRNA will be reduced [32]. Transcripts with short 3′ UTRs show greater stability and usually encode more proteins, as some miRNA binding sites disappear in transcripts with short 3′ UTR [31].

RBPs interact with RNA molecules and affect their splicing, stability, localization, and translation. In addition to competing with miRNAs, RNAs can compete with RBPs. The two regulatory processes are not isolated but are intrinsically linked [26, 32]. RBPs can influence the interactions of ceRNAs by directly occupying RNA target sites or by altering the RNA secondary structure to influence its affinity for miRNAs [24].

In addition, the activity of ceRNAs also depends on the number of MREs contained in each cell [26] and the subcellular localization of ceRNAs [24]. Furthermore, the tissue, disease development and pathological environment also influence the activity of ceRNAs [24]**(**Fig. 1**).**

Exosomes are vesicles encased in a lipid bilayer that can be produced and released by almost all types of cells, including tumor cells. They can contain DNA, mRNA, miRNA, and transport proteins. Li et al. [20] first discovered circRNAs in MHCC-LM3 liver cancer cells and exosomes by RNA-seq. They measured the back-spliced ratio of circular RNA to linear RNA in the exosomes and determined that it was 6-fold higher than the number of cells, indicating that exosomes could be rich in cyclic RNA. In addition, the expression of circ-KLDHC 10 in the serum of patients with colorectal cancer is far higher than that in healthy people, suggesting that circRNAs in tumor exosomes may represent a marker for the clinical diagnosis of cancer.

Because circular RNAs have no open linear tail, they are insensitive to exonucleases and therefore are stably expressed in the cell. Thus, if their expression is not appropriately regulated, they may accumulate in cells. Exosome secretion may thus represent one of the mechanisms for the removal of circular RNAs.

In recent years, increasing evidence has demonstrated that the interactions among ceRNAs, miRNAs, and miRNAs that act downstream of target genes are closely linked to the development of tumors; therefore, this has become a focus in the field of cancer research.

### CircRNAs function as ceRNAs in tumor signaling pathways

Recently, circRNA has been increasingly found to be involved in cellular signaling pathways, such as mitogen-activated protein kinase (MAPK)/extracellular regulated protein kinase (ERK1/2), phosphatidylinositol 3 kinase (PI3K)/protein kinase B (AKT), and Wnt/β-catenin pathways [33–36]. As a relatively upstream regulatory molecule, circRNA acts by releasing downstream molecules by competing with miRNA. Often, a circRNA can regulate several tumors with similar biological properties through competitive binding. To date, circRNA has been found to be involved in several biological processes related to tumor development, including proliferation, apoptosis, and invasion **(**Table 1**)**.

**Table 1:** Circular RNAs function as ceRNAs to regulate and control human cancer progression

Circular RNA	Biological functions	miRNA	Downstream pathways	Tumor types	The expression level	References
CDR1as	proliferation\invasion\ migration\biomarkers	miR-7	EGFR, IGF-IR/PTEN/PI3K/AKT	colon cancer HCC/GC	up	[23, 24]
cir-ITCH	proliferation	miR-7 /miR-214	*ITCH*(Wnt/β-catenin)	lung cancer	down	[26]
circMTO1	proliferation\biomarkers	miR-9	p21	HCC	down	[35]
hsa_circ_0013958	proliferation\biomarkers	miR-134	cyclin D1	lung adenocarcinoma	up	[36]
circRNA_100290	proliferation	miR-29b	CDK6	OSCC	up	[37]
circTCF25	proliferation	miR-103a-3p /miR-107	CDK6	bladder cancer	up	[38]
circPVT1	proliferation\biomarkers	miR-125	E2F2	GC	up	[40]
hsa_circ_001569	proliferation	miR-145	E2F5	colon cancer	up	[41]
circHIPK3	apoptosis	miR-124	IL6R, DLX2	HCC	up	[42]
circUBAP2	apoptosis\biomarkers	miR-143	Bcl-2	myeloma	up	[50]
hsa_circ_0001982	apoptosis\biomarkers	miR-143	Bcl-2	breast cancer	up	[51]
circCCDC66	apoptosis	miRNA-33b /miR-93	MYC	colon cancer	up	[52]
circ_0009910	apoptosis\biomarkers	miR-449a	IL6R	osteosarcoma	up	[53]
circGFRA1	apoptosis\biomarkers	miR-34a	GFRA1	TNBC	up	[55]
hsa_circ_0005986	Invasion/ migration	miR-129-5p	Notch1 mRNA	HCC	down	[60]
circRNA-MYLK	Invasion/ migration	miR-29a	VEGFA	bladder cancer	up	[62]
circ-TTBK2	Invasion/ migration	miR-217	HNF-1β	glioma	up	[63]
circular RNA SRY	Invasion/ migration	miR-138	RhoC	bile duct	up	[2]
circ-0016347	Invasion/ migration	miR-214	caspase-1	osteosarcoma	up	[70]
hsa_circ_0005075	clinical diagnosis biomarkers			HCC	up	[72]

#### CircRNA and the signaling pathways involved in cell proliferation

Near-limitless multiplication capacity is one of the important characteristics of tumor cells [37]. The regulation of cell proliferation is a complex and precise process driven by a variety of signals that cause the cell to divide and grow by regulating the cell cycle. Consequently, disorders affecting cell cycle regulation play an important role in the excessive proliferation of tumor cells [38]. Many studies have shown that circRNAs function as ceRNAs in the control of tumor proliferation.

Some circRNAs have been reported to affect epidermal growth factor (EGFR)/RAF1/MAPK signaling. MAPK/ERK, which participates in cell proliferation, can control mitosis by signal amplification [39]. CDR1as/ciRS-7, which acts as a competitive miR-7 sponge and inhibits its function, can release insulin growth factor 1 receptor (IGF-IR) and other molecules and affect the MAPK/ERK1/2 and PI3K/AKT intracellular signaling pathways [33, 40, 41]. As a result, CDR1as is involved in the development of colon cancer, gastric cancer, and hepatocellular carcinoma by controlling proliferation. Several studies have demonstrated that overexpression of CDR1as in tumor cell lines results in active tumor proliferation [16, 33, 40, 42, 43]. Similarly, cir-ITCH plays a similar role in lung cancer by competitively binding to miR-7 [36].

**Fig. 2:**
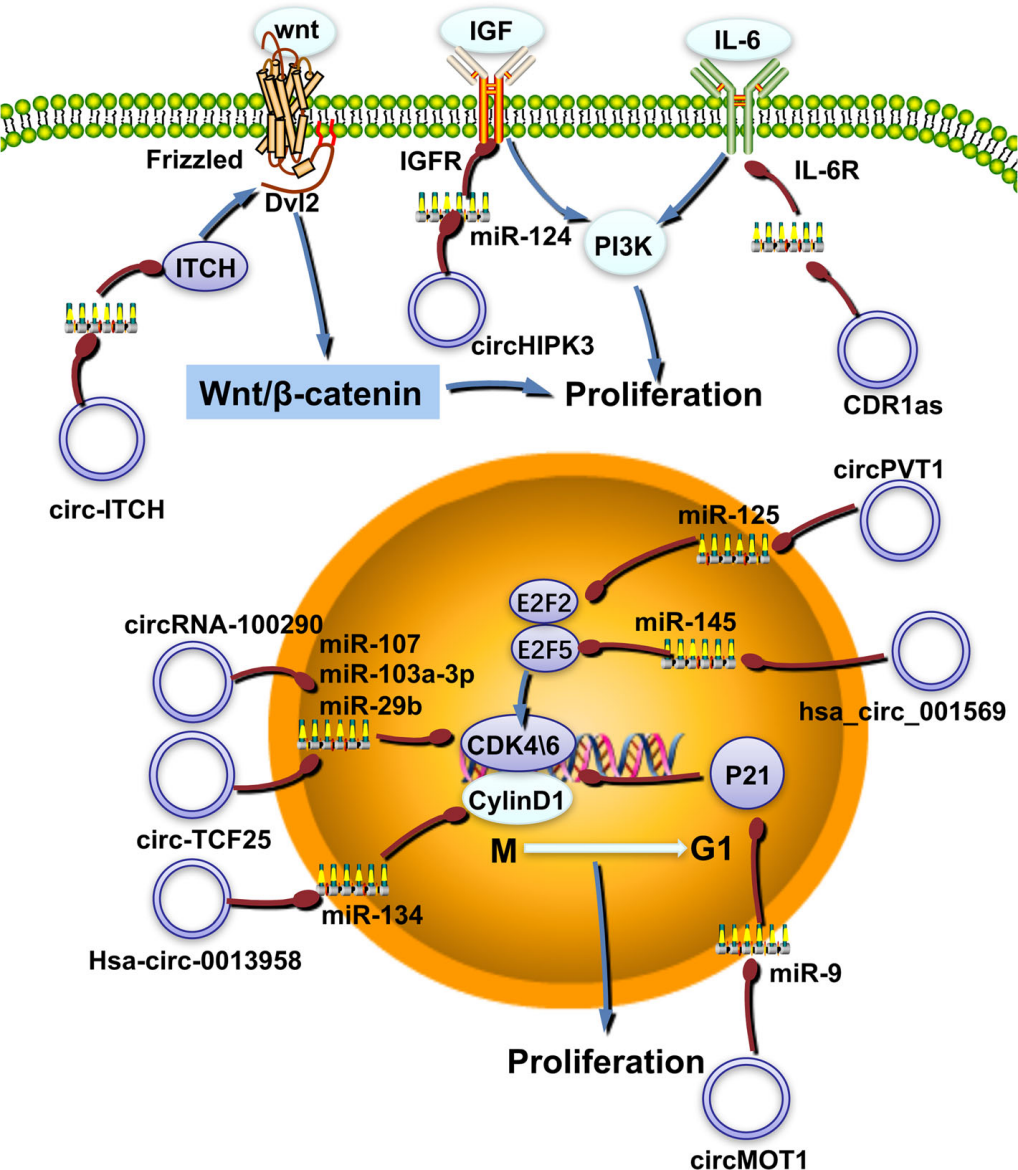
circRNAs function as ceRNAs in regulating tumor cell proliferation. The regulation of the cell cycle is an important part of cell proliferation. CDR1as, circMTO1, circRNA_100290, circTCF25, hsa_circ_0013968, circPVT1, and hsa_circ_001569 participate in the regulation of the cell cycle through related molecules, thus modulating cell proliferation. At the same time, signaling pathways such as Wnt/β-catenin and MAPK also play an important role in the proliferation of tumor cells; cir-ITCH and other circRNAs play a regulatory role in these signaling pathways

Some circRNAs can regulate cell proliferation by targeting key molecules involved in the regulation of the cell cycle. p21 is a negative regulator of the cell cycle and a member of the cyclin-dependent kinase 1 family (CDK1); in association with cyclin, cyclin-dependent kinases (CDKs) or cyclin-CDKs, p21 induces cell cycle arrest and blocks cell proliferation [44]. Furthermore, when the expression of circMTO1 in liver cancer cells is decreased, the bound miR-9 is released, resulting in decreased p21 gene expression and a significant increase in tumor proliferation [45]. CircRNAs are also involved in the positive regulation of the cell cycle. Hsa_circ_0013958 in lung adenocarcinoma can competitively bind miR-134 and upregulate cyclin D1, leading to an increase in cell proliferation [46]. CircRNA_100290 and circTCF25 can bind miR-29b and miR-103a-3p/miR-107, which results in increased levels of CDK6, thus increasing tumor proliferation [47, 48]. The E2F transcription factors control the cell cycle by regulating the genes required to cross the G1/S DNA synthetic gap. Due to the key role played by cell cycle anomalies in the proliferation of cancer, E2Fs are closely associated with tumor prevalence [49]**(**Fig. 2**)**. In addition, circPVT1 and hsa_circ_001569 compete endogenously by binding to target miRNAs and increase the levels of E2F2 and E2F5, thus, enhancing tumor proliferation [50, 51].

Some reports have found that circRNA can also regulate cell proliferation by regulating immune molecules. Qiupeng Zheng’s team at Fudan University found that overexpression of circCHIPK3 in liver cancer increased the expression of the interleukin 6 receptor (IL6R) by inhibiting the expression of miR-124, which resulted in tumor proliferation [52]. IL6R is bound by its IL-6 ligand and can activate downstream signaling pathways, such as MAPK, PI3K, and signal transducer and activator of transcription 3 (STAT3), which promotes tumor cell proliferation [53–55].

CircRNA can also regulate the Wnt/β-catenin signaling pathway, resulting in the proliferation of tumor cells. The Wnt/β-catenin signaling pathway in turn regulates the pluripotency of cells during development and plays a role in the determination of cell fate [56].

Cir-ITCH increased *ITCH* levels by inhibiting miR-7 and miR-214, which inhibited the activity of disheveled segment polarity protein 2 (Dvl2). Because Dvl2 is an important activator of the Wnt/β-catenin pathway [57], this resulted in inhibition of the signaling pathway [36] **(**Fig. 2**)**.

#### CircRNAs and apoptosis-associated signaling pathways

The loss of apoptosis is another important characteristic of tumors [37]. Recent studies have found that some circRNAs are involved in the regulation of apoptosis in tumor cells.

**Fig. 3:**
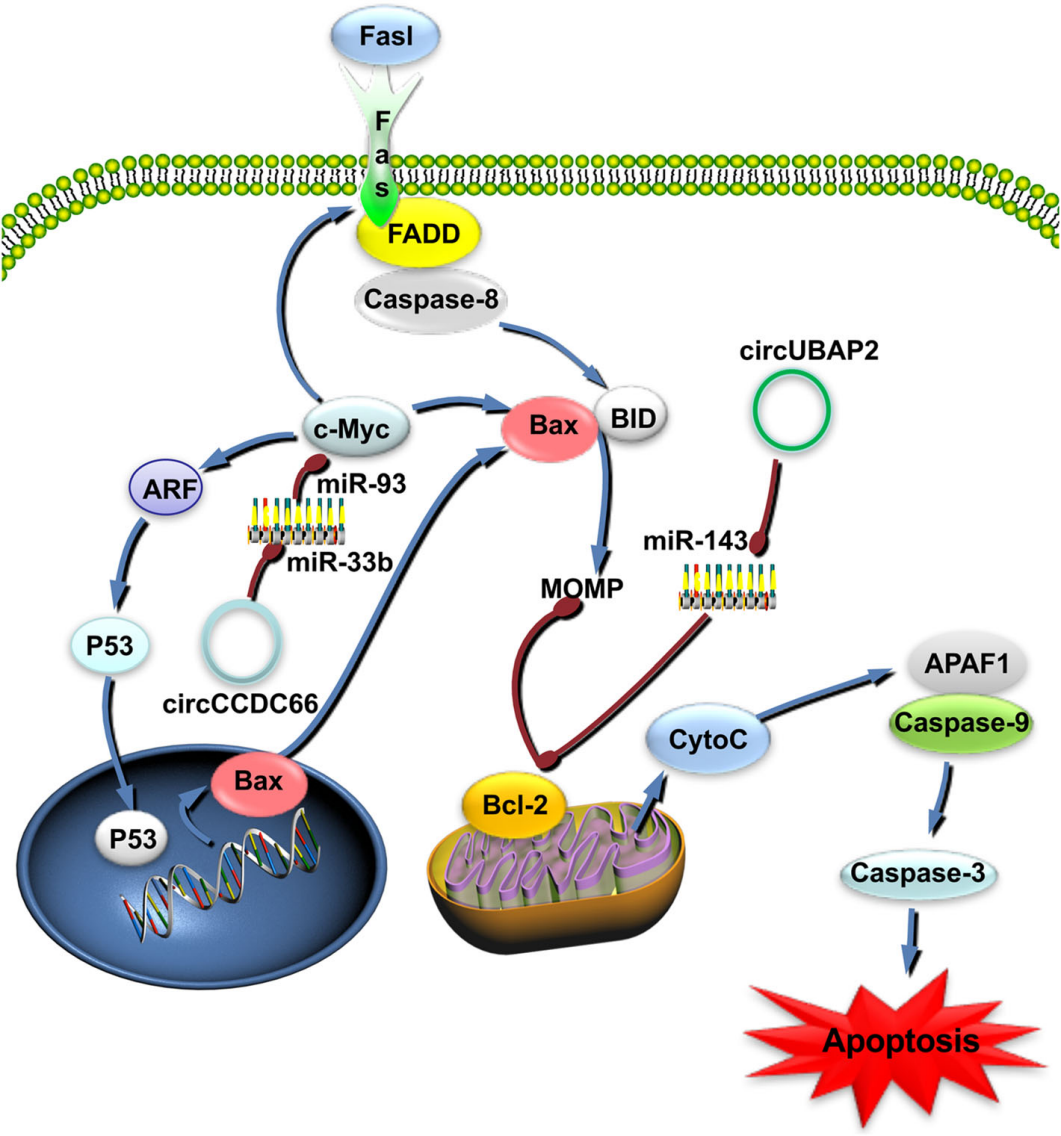
circRNAs function as ceRNAs in the regulation of apoptosis in tumor cells. The expression of c-MYC affects apoptosis. One pathway consists of stabilizing p53 through ARF, whereas a second pathway involves a mechanism independent of the apoptotic pathway involving FasL and DNA damage [2]; the mechanism of kissing apoptosis involves the activation of the Bcl2-associated X protein (BAX), thereby promoting the release cytochrome C from mitochondria and triggering apoptosis. In this signaling pathway, circRNAs also function as ceRNAs. CircCCDC66 can promote apoptosis by inhibiting miR-93, thereby promoting the expression of c-MYC. However, circUBAP2 can control cancer by reducing Bcl-2 levels as a consequence of sequestering miR-143

The Bcl-2 family of proteins plays a key role in the apoptotic pathway. The anti-apoptotic protein Bcl-2 is located in the outer membrane of the mitochondria and inhibits the release of apoptotic factors such as cytochrome C, thus inhibiting apoptosis [58]. Moreover, Bcl-2 may represent a target for miR-143 [59]. The endogenous circUBAP2 and hsa_circ_0001892 both compete to inhibit the activity of miR-143 and reduce apoptosis in myeloma and osteosarcoma cells [60, 61].Moreover, it has been reported that circCCDC66, which is overexpressed in lung adenocarcinoma, can inhibit tumor apoptosis by regulating the MYC oncogene. miRNA-33b and miR-93 could both regulate the MYC gene and promote apoptosis in tumors, whereas circCCDDC66 could inhibit this process by inhibiting both miRNAs [62] **(**Fig. 3**)**.

Studies have shown that circ_0009910 acts as a sponge for miR-449a and promotes the expression of IL6R, which is the target of miR-449a. Moreover, circ_0009910 plays a role in inhibiting apoptosis in tumors [63]. Previous reports have indicated that IL6R may activate the STAT3 signaling pathway [64]. In a similar manner, circHIPK3 can also inhibit apoptosis by exerting an endogenous competitive effect and regulating the expression of IL6R [52] **(**Table 1**)**.

CircRNAs as ceRNAs regulate the expression of GDNF family receptor alpha-1 (GFRA1) by regulating the levels of miR-34a, and they have an anti-apoptotic function in triple-negative breast cancer [65]. GFRA1 is a cell-surface receptor for glial cell-derived neurotrophic factor (GDNF), which is reported to have an anti-apoptotic effect [59, 66] **(**Table 1**)**.

#### CircRNAs are involved in invasion and migration as ceRNAs

Invasion and migration are indispensable characteristics of malignant tumors [37]. The epithelial-mesenchymal transition (EMT) is an important process regulating early invasion and migration in many tumors [67]. Wnt, TGFβ, and Notch ligands play key roles in EMT. Novel advances investigating the interplay between EMT and tumors have revealed several molecules, such as epidermal growth factor (FGF), insulin growth factor (IGF), and other hepatocyte growth factors (HGF) involved in EMT in tumors [68]. MiRNAs are involved in the regulation of EMT as non-coding RNAs [69]. As a sponge for miRNAs, circRNAs can also participate in the regulation of EMT through endogenous competition mechanisms, which can affect the invasion and migration of tumors **(**Table 1**)**.

**Fig. 4:**
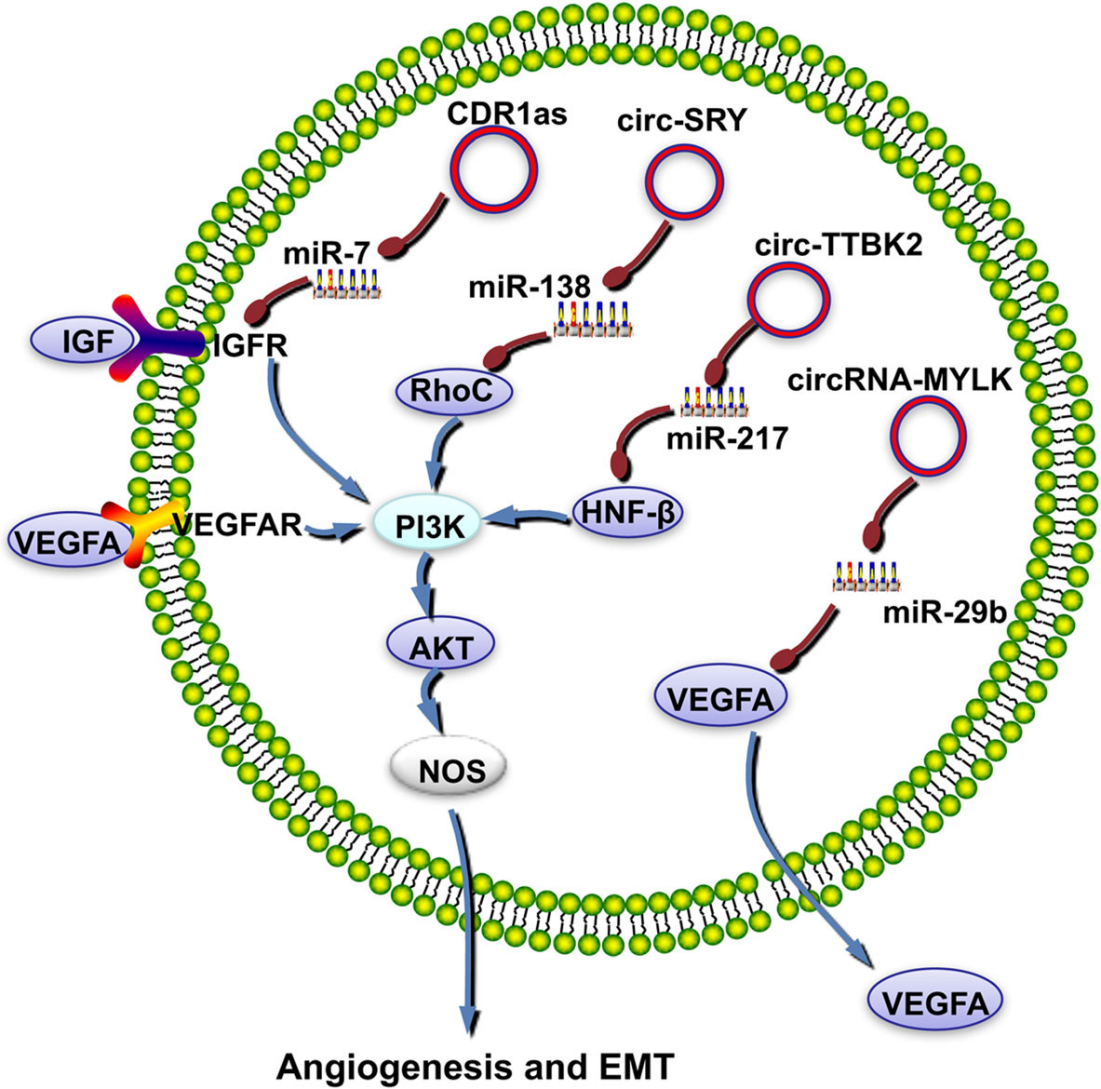
circRNAs function as ceRNAs in regulating tumor invasion and migration. circRNAs regulate the invasion and migration of multiple factors in tumor cells, including RhoC, Notch, VEGFA, IL6R, and HNF-1β. These factors all participate in stimulating PI3K, which can activate AKT. This can promote angiogenesis and the epithelial-to-mesenchymal transition. circRNAs function as ceRNAs in this regulation

The highly expressed hsa_circ_0005986 was found to competitively bind miR-129-5p, resulting in decreased expression of Notch1 mRNA; this led to the inhibition of EMT with an accompanying anti-cancer effect [70]. However, low expression of hsa_circ_0005986 in liver cancer led to increased invasion and migration of the tumor cells. CDR1as exhibits a variety of regulatory functions, in addition to regulating the proliferation of tumor cells. For example, it can also promote tumor invasion and migration by inhibiting miR-7 and regulating EGFR and IGF-IR [33, 34, 41] **(**Fig. 4**)**.

VEGFA is an important angiogenic factor involved in EMT [71]. High expression of circRNA-MYLK in bladder cancer can increase the expression of VEGFA mRNA by binding miR-29a as an RNA sponge, leading to enhanced tumor cell migration [72]. In glioma, circ-TTBK2 can bind and inactivate miR-217, leading to activation of the PI3K/AKT and ERK pathways via HNF-1β, which has a miR-217 binding site in its 3′ UTR. This promotes the attack of glioma cells, resulting in a high degree of tumor malignancy [73]. Circular SRY is a specific circular transcript present in the testicles, and it is expressed from a male sex determination gene [74, 75].Circular SRY binds to miR-138 and inhibits its activity [2]. MiR-138 SRY regulates the Ras homolog gene family member C (RhoC) mRNA. Because the expression of RhoC is regulated bymiR-138, its translation increases, thus promoting the proliferation, migration, and invasion of cholangiocarcinoma cells [2, 76] (Fig. 4). Whether circular SRY can be used as a ceRNA to inhibit miR-138 and regulate RhoC needs further discussion. The formation of the tumor microenvironment is beneficial for tumor invasion. Caspase-1 can activate the pro-inflammatory cytokines IL-1β and IL-18, and promote inflammation and the formation of the tumor microenvironment, thus potentially promoting tumor infiltration and metastasis [77–79]. Circ-0016347 can competitively bind miR-214 and remove the inhibition of caspase-1 by miR-214, thus increasing caspase-1 levels. Therefore, through the regulation of circ-0016347, tumor invasion and metastasis are enhanced [80] (Table 1).

### circRNA and exosomes

Exosomes are vesicles encased in a lipid bilayer that are produced and released by almost all types of cells, including tumor cells. They store DNA, mRNA, miRNAs, and transport proteins. Li et al. [81] first discovered circRNAs in MHCC-LM3 liver cancer cells and exosomes by RNA-seq. They measured the back-spliced ratio of circular RNA to linear RNA in the exosomes and determined that it was 6-fold higher than the number of cells, indicating that exosomes could be enriched in cancer [81].

Because circular RNAs have no open linear tail, they are insensitive to exonucleases and therefore are stably expressed in the cell [81, 82]. Thus, if their expression levels are not regulated, they may accumulate in cells. Exosome secretion may thus represent one of the mechanisms for the removal of circular RNAs. Another possibility is that circRNAs may regulate cell communication through exosomes in some cases [83]. ciRS-7 /CDR1, which can be used as a sponge for miR-7, is present in exosomes. miR-7 upregulation in cells is associated with significantly reduced CDR1 levels in exosomes. However, the CDR1 levels in cells change very little, suggesting that the role of circRNAs in exosomes may be to transferring miRNA between different cells [81, 83].

### CircRNAs as novel biomarkers in cancer

In contrast to linear RNAs, the 3′ and 5′ ends of circRNAs are not exposed. Therefore, they are not sensitive to ribonucleases, such as exonucleases, and are more stable [84]. Some circRNAs are specific for certain tumors. CircRNA with high expression in cancer tissues of some cancer patients, such as hsa_circ_0013958, also have high levels in plasma [46]. This means that detection can be more convenient. Additionally, the use of RNA sequencing and qRT-PCR has made the detection of circRNAs faster and more convenient, making circRNAs easier to extract and detect than proteins. Therefore, circRNAs represent an ideal biomarker for the prognosis and diagnosis of cancer. Recent studies have found that the expression profiles of circRNAs are changed in a variety of cancers. One study examined the levels of circCCDC66 in nontumor tissues (*N*; *n* = 76), polyp tissues (*P*; *n* = 22) and tumor tissues (T; *n* = 131) in patients with colorectal cancer and found that the levels of circCCDC66 were significantly higher in cancer tissues, whereas the prognosis of patients who displayed higher expression of circCCDC66 was worse. An area under the curve analysis was then used to show that the circCCDC66 levels in 88 patients with colorectal cancer who had been randomly selected was higher than that in randomly selected normal subjects. This demonstrated that circCCDC66 is a good marker for the diagnosis and prognosis of colon cancer [62]. Moreover, the expression of hsa_circ_0005075 in hepatocellular carcinoma was elevated and closely linked to clinical features such as tumor size [85]. It was also observed that circPVT1 could be used as a prognostic marker for gastric cancer, as high expression of circPVT1 was correlated with a worse prognosis [50]. Due to the high expression of CDR1as in various cancers, the expression of CDR1as may not be an obvious specific marker for early cancer diagnosis. Using a follow-up survey, it was found that other circRNAs, such as circMTO1, hsa_circ_001395,circUBAP2, circCCDC66,circGFRA1, hsa_circ_0004277 and hsa_circ_0005986, are also valuable markers for cancer diagnosis and prognosis [45, 46, 60, 62, 65, 86]**(**Table 1**)**. CircRNAs are more stable in tissues, serum, and urine because of their characteristics. The development of high-throughput sequencing has removed the obstacles to identifying circRNAs. However, circRNAs have not been clinically used thus far. A complete and reliable standard for diagnostic or prognostic markers remains to be further explored. However, the advantages of circRNA as biomarkers are becoming increasingly clear, and they may provide a new generation of tumor molecular biomarkers.

### The role of circRNA as a ceRNA in tumor immunity

Tumor cells have immunogenicity, but tumors can evade immune surveillance [37]. In fact, throughout the course of tumor development, tumor cells must overcome the barrier of internal and external immunity to become a tumor. Only when cancerous cells escape immune surveillance and overcome immune function through various immune evasion mechanisms can the tumor progress or kill the host. CircRNA has rich potential to regulate tumor immunity because of its innate characteristics [87].

It has been reported that circRNA can be used as ceRNA to help the tumor evade immune surveillance. Inflammation in the tumor microenvironment is closely related to tumor immunity. Therefore, the study of signal transduction pathways for tumor-related inflammation will help to reveal the relationship between immunity and tumor development [87, 88]. As we discussed earlier, circRNA can be used as ceRNA to participate in the expression of inflammatory mediators. CircHIPK3 inhibits the activity of mir-124 and promotes the expression of IL-6R by binding miR-124 [52]. Whether circHIPK3 can affect the tumor immune response by promoting IL-6R remains to be further explored. The aforementioned hsa_circ_0020397 binds to miR-138 to inhibit the activity of miR-138 and promotes the expression of miR-134, which in turn regulates molecules such as telomerase reverse transcriptase and PD-L1 in colorectal cancer cells [66]. As a result of the moderate or high expression of hsa_circ_0020397 in CRCC, PD-L1 is up-regulated and can interact with PD-1 to enable cancer immune escape [89]. A recent study indicated that circAmotl1 up-regulates Dnmt3a, which could lead to methylation of the miR-17 promoter and reduce the expression of miR-17-5p [87, 90]. Finally, STAT3 expression is increased and plays an important role in tumor-mediated immunosuppression [90].

### Perspectives

With the rapid development of next-generation sequencing technology and bioinformatics, circRNAs are being increasingly identified. Extensive software is available for the identification of circRNAs based on second-generation sequencing data. Software based on comparison strategies or DeNOVO assembly is also currently present. In this field, the major issues are as follows. 1. Accuracy and false positive rate. Second-generation sequencing uses short sequences, the original data features are fuzzy, and the accepted depth of the sequence is often calculated by the likelihood algorithm in comparison, which leads to relatively low quantitative accuracy because of complex variable shear events [91]. In addition, due to the limited understanding of the cycling mechanisms, the current algorithm has a higher false positive rate and needs to be improved [92]. 2. Resource appropriation. The cyclization mechanism is a complex variable shear event, which requires a large number of parameters to adjust the operation; therefore, it occupies a considerable amount of resources and the operation speed is slow [92]. At present, the problems caused by data characteristics cannot be solved, but we can improve the understanding of the cyclization mechanism and improve the test to reduce the false positive rate. Resource consumption can also be decreased through development of new algorithms.

At least two mechanisms exist for the formation of mature circRNA molecules, which are different from the selective splicing of linear RNA [4]. Although having the same cis-elements, expression of circRNAs from the same loci varies in different cell lines and tissues [93–95]. A recent study showed that nuclear factor 90 (NF90) and its isoform NF110, which are induced by human interleukin enhancer binding factor 3 (ILF3), could promote circRNA production [96]. DHX9, an abundant nuclear RNA helicase, binds specifically to inverted-repeat Alu elements that are transcribed as parts of genes. Loss of DHX9 leads to an increase in the number of circular-RNA-producing genes and amount of circular RNAs [97]. We predict that more of these molecules are involved in the regulation of circRNA production.

The current study found that circRNAs generally act as non-coding RNAs and mainly function as miRNA sponges, regulating transcription and participating in protein interactions. However, recent studies have found that circ-FBXW7 can encode a new protein of 21 kDa [98], whereas circ-ZNF609 also encodes a functional protein. This suggests that circRNAs can encode functional proteins, but it remains to be further explored whether this represents a primary function of these RNA molecules. Current data indicate that circRNA, as a ceRNA, can regulate tumor proliferation, apoptosis, and angiogenesis. We predict that circRNAs may play an important role in the regulation of other tumor characteristics, and thus may form a detailed and complex regulatory system.

## Conclusions

An increase or decrease in circRNA expression can affect the biological characteristics of tumor cells. Research aimed at uncovering the mechanism underlying these effects has shown that circRNAs serve as ceRNAs to mediate miRNA functions and may serve as a target for cancer treatment. In the future, targeting of circRNA may be used not only for direct anti-cancer effects, but also in immunotherapy. Recent studies have shown that purified circRNA activates RIG-1, which has an immune effect, and then activates innate immunity against tumors [99, 100]. In addition, it has been suggested that circRNA can mediate cell communication between tumor immune components, as well as serve as a new tumor antigen, thus providing a potential target for immunotherapy [87]. However, how to effectively modulate circRNA levels in target cells remains an unresolved issue. At present, no tumor suppressor drug exists to target circRNAs. However, the roles and characteristics of circRNAs suggest that they have great potential for cancer treatment.

## Abbreviations

AKT: Protein kinase B
BAX: Bcl2-associated X protein
CDK: Cyclin-dependent kinase
ceRNA: Competing endogenous RNA
circRNA: Circular RNA
Dvl2: Disheveled segment polarity protein 2
EGFR: Epidermal growth factor
EMT: Epithelial-mesenchymal transition
ERK1/2: Extracellular signal-regulated kinases ½
FGF: Epidermal growth factor
GDNF: Glial cell-derived neurotrophic factor
GFRA1: GDNF family receptor alpha-1
HCC: Hepatocellular carcinoma
HGF: Hepatocyte growth factor
IGF: Insulin growth factor
IGF-IR: Insulin growth factor 1 receptor
IL6R: Interleukin 6 receptor
lncRNA: Long non-coding RNA
MAPK: Mitogen activated protein kinase
miRNA: microRNA
MREs: miRNA response elements
PI3K: Phosphatidylinositol 3 kinase
RBPs: RNA binding proteins
UTR: Untranslated region
